# Significance of immunogenic cell death-related genes in prognosis prediction and immune microenvironment landscape of patients with cutaneous melanoma

**DOI:** 10.3389/fgene.2022.988821

**Published:** 2022-09-21

**Authors:** Weijiang Fu, Guangxin Ma

**Affiliations:** Hematology and Oncology Unit, Department of Geriatrics, Qilu Hospital of Shandong University, Jinan, China

**Keywords:** cutaneous melanoma, immunogenic cell death, risk model, immune infiltration, prognostic value

## Abstract

Cutaneous melanoma (CM) is one of the most life-threatening tumors. Although targeted therapies and immune checkpoint inhibitors have significantly improved patient outcomes over the past decades, they still have their efficacy limitations. Immunogenic cell death (ICD) induces regulated cell death through immunogenic signal secretion and exposure. Accumulated evidence suggests that the ICD process is an effective target for the treatment of a variety of tumor types, including CM. However, the research on ICD in CM is far from complete, and its clinical value has not been widely concerned. By analyzing the Cancer Genome Atlas (TCGA) database, we constructed a new risk model based on 4 ICD-related genes and validated its ability to predict the prognosis of CM patients. In addition, we comprehensively analyzed the tumor microenvironment (TME) of CM patients and showed a significant immunosuppressive TME in the high-risk group compared with the low-risk group. By Immunophenoscore (IPS), we further explored the correlation between the model and immunotherapy response. The data of Genomics of Drug Sensitivity in Cancer (GDSC) database were further extracted to analyze drug sensitivity and evaluate its correlation with the established risk model. In the end, differential expressed genes (DEGs) were analyzed by Gene Set Variation Analysis (GSVA) to preliminarily explore the possible signaling pathways related to the prognosis of ICD and CM. The results of this study provide new perspectives and insights for individualized and accurate treatment strategies for CM patients.

## Introduction

As one of the threatening types of cancer, cutaneous melanoma (CM) accounts for 10% of all new skin cancer cases diagnosed, and its prevalence and mortality are further increasing worldwide (Siegel et al., 2020). Due to its high rate of invasion and distant metastasis, CM accounts for 72% of skin cancer deaths (Schadendorf et al., 2018). In recent years, immune checkpoint blockade has attracted extensive attention for its remarkable efficacy in clinical application of melanoma (Sullivan and Flaherty, 2015). Despite significant advances in targeted therapies and novel immunotherapies (Davis et al., 2019; Steeb et al., 2020), the efficacy of all treatments is greatly affected in comparison to aggressive surgical treatment in the early stages of the disease. Therefore, there is a need to identify tumor-related biomarkers and stages that influence prognosis. Therefore, accurate early diagnosis is crucial for a good prognosis of melanoma.

Immunogenic cell death (ICD) is a type of regulated cell death with different functions, which is characterized by the secretion and exposure of immunogenic signals in dead tumor cells. Immunogenic signals are known as damage related molecular patterns (DAMP) (Tesniere et al., 2008; Bezu et al., 2015). These DAMPs include: an endoplasmic reticulum (ER) partner exposed to the plasma membrane of dead cells, calreticulin (CALR), which is conducive to the function of phagocytes (Obeid et al., 2007); ATP secreted in an autophagy-dependent manner during ICD eventually plays a chemotactic role (Elliott et al., 2009); under the action of ICD, cells release a nucleoprotein high mobility group box 1 (HMGB1) that binds to toll like receptor 4 (TLR4), which acts as adjust like effects (Apetoh et al., 2007); type I interferon (IFN) is secreted during ICD through interferon expressed on cancer and immune cells α and β Receptors ultimately mediate chemotaxis and immune stimulation (Sistigu et al., 2014). ICD and its related damp have been reported to affect the outcome of a variety of tumor diseases. The ICD process of damp above will lead to the secretion of immunogenic signals in tumor cells, which can activate dendritic cells (DC) and change immunosuppression in tumors (Radogna and Diederich, 2018). Additionally, chemotherapeutic drugs cause ICD, which in turn enhances the anti-tumor immune response (Zhao et al., 2016). Previous studies have reported that radiotherapy and some chemotherapy drugs (such as Adriamycin and oxaliplatin) can induce ICD *in vitro* and *in vivo* and stimulate the immune response against tumor cells (Paolini et al., 2011; Pol et al., 2015). The accumulated preclinical and clinical evidence shows that the ICD process is a promising effective therapy target for a variety of tumors, including CM (Sethuraman et al., 2020; Zhang et al., 2020). However, the clinical value of ICD in CM has not been widely concerned.

In this study, after analyzing the Cancer Genome Atlas (TCGA) database, we systematically studied the relationship between ICD related genes and the clinicopathological characteristics of CM patients. Based on 4 ICD related-genes, we constructed a new risk model and verified its ability to predict the prognosis of CM patients. In addition, we comprehensively analyzed the immune microenvironment of CM patients, further explored the correlation between the model and immune response and drug sensitivity treatment, and preliminarily explored the potential signal pathways in the process. The results of this study provide new perspectives and insights for the individualized and accurate treatment strategies of CM patients.

## Results

### Identification of prognostic immunogenic cell death-related genes

**FIGURE 1:**
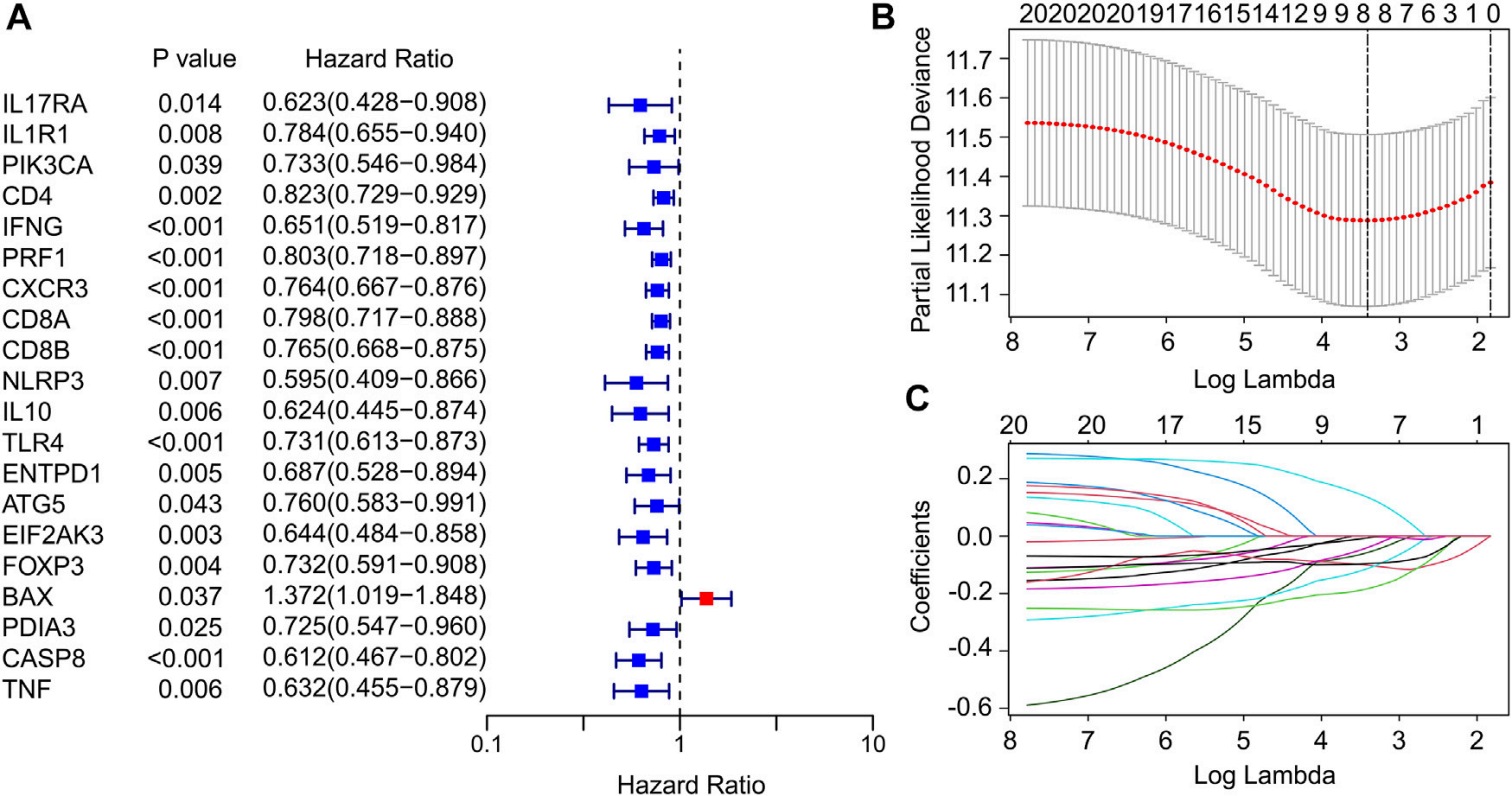
Identification of prognostic ICD-related genes in CM. **(A)** Univariate Cox regression analysis suggests that 20 ICD-related genes are associated with OS in CM. **(B,C)** Least absolute shrinkage and selection operator analysis (LASSO) shows the minimal lambda and coefficients of prognostic ICD-related genes.

In this study, a total of 33 ICD genes were extracted to evaluate the prognosis signature of patients with CM. According to the univariate Cox regression analyses, 20 ICD genes associated with overall survival (OS) were identified (Figure 1A). The 20 ICD genes were subsequently subjected to the least absolute shrinkage and selection operator (LASSO) model to calculate the optimal coefficients, and 8 ICD-related genes were selected for the subsequent analysis (Figure 1B,C). The expression of ICD genes in normal tissues and tumor tissues was statistically analyzed in Supplementary Figure S1.

### Risk model construction of immunogenic cell death-related genes

**FIGURE 2:**
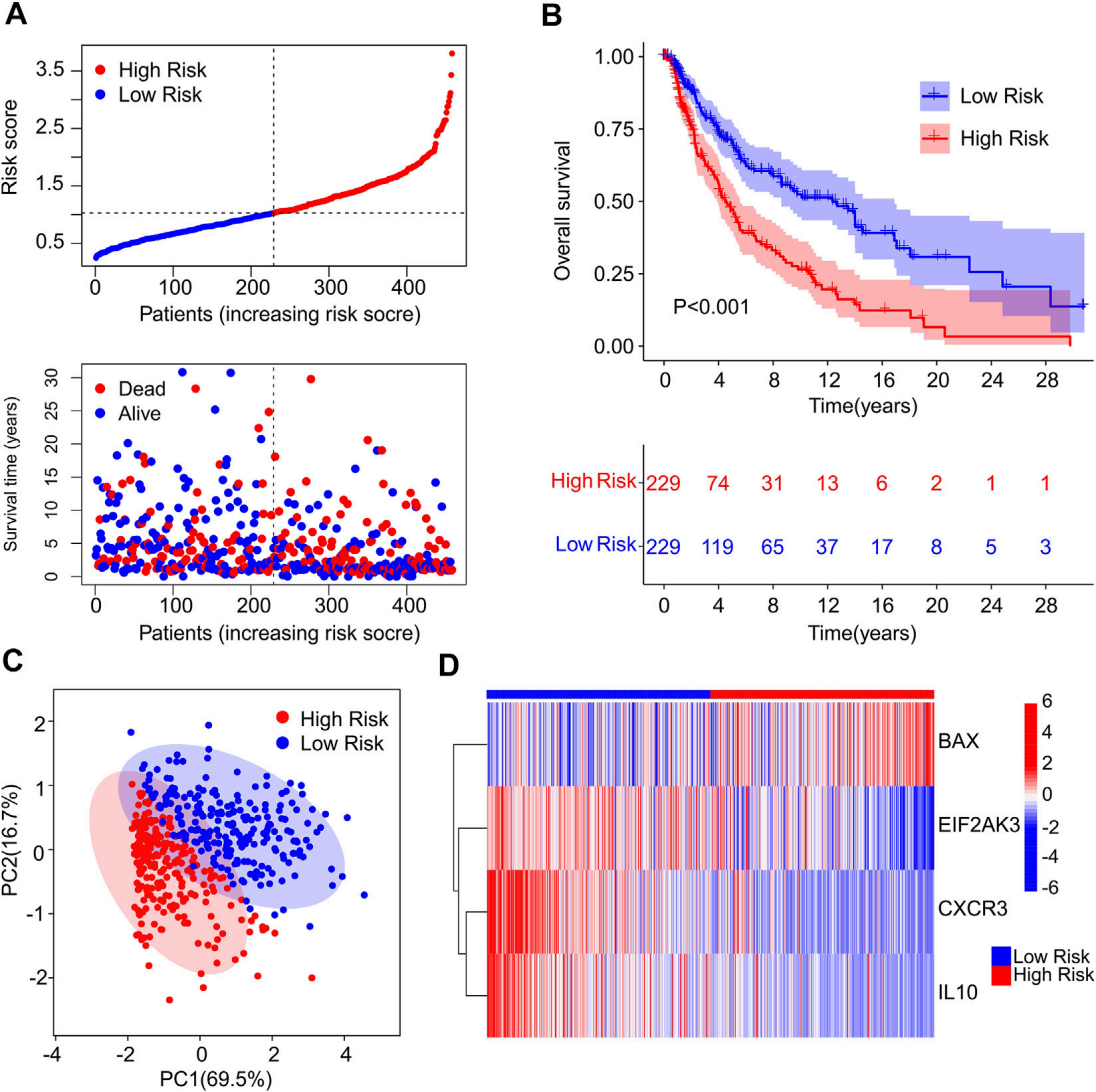
Construction of risk model based on the prognostic ICD genes of patients with CM. **(A)** Distribution of risk score of CM patients and scatter dot plot shows the correlation of risk score and survival time. **(B)** The Kaplan-Meier survival curve shows that the OS of low-risk group was significantly higher than high-risk group. **(C)** Principal component analysis (PCA) shows a significant difference in high- and low-risk group based on the four prognostic ICD genes. **(D)** Heatmap illustrates the expression of four prognostic ICD genes (*BAX, EIF2AK3, CXCR3* and *IL-10* in high- and low-risk groups.

A fresh risk model was established to evaluate the prognosis of CM patients based on the ICD-related genes prognostic signature. Based on the multivariate Cox regression analysis, 4 ICD-related genes including *BAX, EIF2AK3, CXCR3* and *IL10* were identified to construct the risk model. According to the median of risk score, the patients with CM were ranked with the risk score and classified into low- and high-risk group. The scatter dot plot showed that the survival time of CM patients was inversely correlated with the risk score (Figure 2A). The Kaplan-Meier survival curve suggested that the OS rate of CM patients in low-risk group was significantly longer than those in high-risk group (Figure 2B). Principal component analysis (PCA) results illustrated a clear separation between low- and high-risk groups based on the 4 prognostic ICD-related genes (Figure 2C). Heatmap visualization results revealed the expression differences of 4 ICD-related genes in low- and high-risk group (Figure 2D). The low-risk groups showed a lower expression of *BAX*, whereas the expression of *EIF2AK3, CXCR3*, and *IL10* were higher in low-risk group. These results demonstrate that the risk model construction based on the prognostic signature of 4 ICD-related genes could accurately evaluate the prognosis of CM patients.

### Constructing a risk mode in the Cancer Genome Atlas and GEO cohort

**FIGURE 3:**
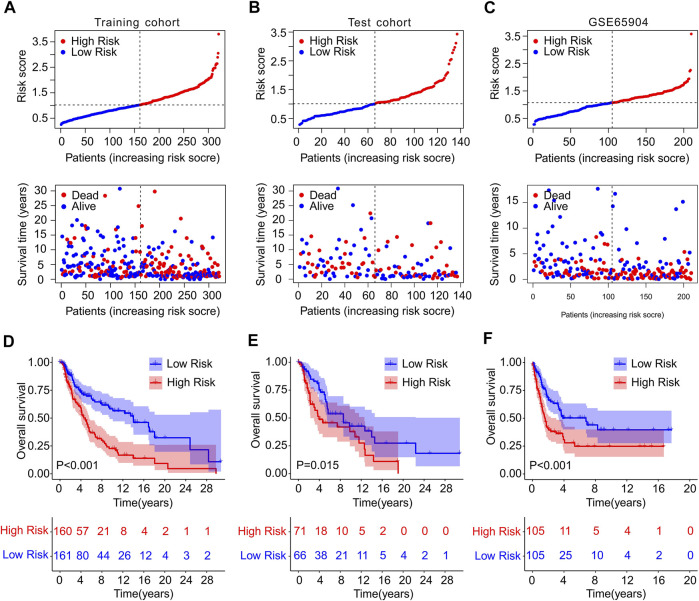
Risk model construction in training cohort and test cohort and GSE65904. The distribution of risk score calculated by ICD-related genes prognostic signature and the scatter dot plot shows the association of risk score and survival time in **(A)** Training cohort, **(B)** test cohort, **(C)** GSE65904. **(D–F)** The Kaplan-Meier survival curve displayed the OS rate of patients with CM in low- and high-risk group in training cohort, test cohort, and GSE65904.

To confirm the accuracy and reliability of the prognosis value of ICD-based risk score, a risk model was constructed using TCGA and GEO cohort. The patients with CM in TCGA cohort were randomly divided into training and test cohort based on the 4 ICD-related genes prognostic signature. The CM patients were ranked according to the median risk score in both cohorts, and the scatter dot plot revealed that the survival time of CM was inversely associated with risk score (Figures 3A,B). The PCA analysis of the training set and validation set was shown in Supplementary Figure S2.

Meanwhile, GSE65904 cohort was employed to further investigate the precision of risk model. According to the median risk score, the CM patients in GEO cohort were ranked and the scatter dot plot indicated a significant correlation of survival time and risk score (Figure 3C). The Kaplan-Meier survival curve analysis showed that patients in the training cohort with low-risk score had higher OS rate than those with high-risk score (*p* < 0.001, Figure 3D). Additionally, the OS rate of CM patients with low-risk score was significantly higher compared to those with high-risk score in the test cohort (*p* = 0.015, Figure 3E). The result of GSE65904 cohort illustrated that the OS rate of patients with CM in low-risk group was significantly longer than high-risk group (*p* < 0.001, Figure 3F). Collectively, these above findings demonstrate that constructing the risk model based on the 4 ICD-related genes prognostic signature is accurate and reliable.

### Risk model based on the immunogenic cell death-related genes is an independent prognostic indictor

**FIGURE 4:**
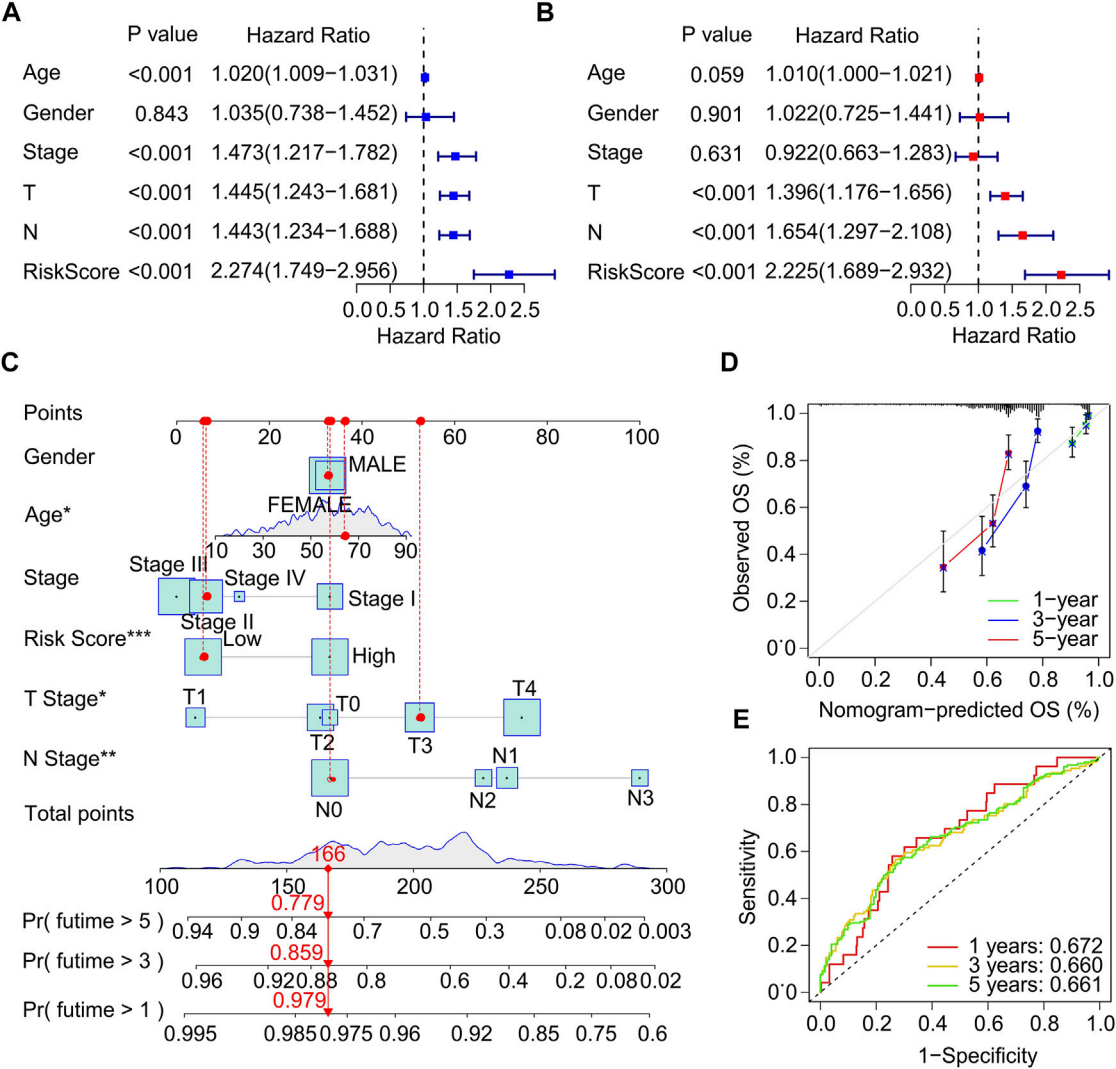
Independent prognostic analysis of clinical characteristics and risk score. **(A)** Univariate Cox regression analysis suggests a clear association between OS rate and clinical characteristics including age, gender, stage, T stage, N stage, and the risk score. **(B)** Multivariate Cox regression analysis indicates that T stage, N stage and risk score are an independent prognostic indicator for CM. **(C)** Nomogram construction of risk score and clinicopathological characteristics to predict the 1-, 3-, 5-years OS rate of CM patients. **(D)** Calibration curve shows the accuracy between predictive capacity and actual OS rate of 1-, 3-, and 5-years. **(E)** Time-dependent ROC curve shows the AUC at 1-, 3-, and 5-years.

Univariate and multivariate Cox regression analysis were performed to investigate the ICD-related genes prognostic signature was an independent prognosis factor for CM. Univariate Cox regression analysis suggested that age (hazard ratio (HR) = 1.020, *p* < 0.001), stage (HR = 1.473, *p* < 0.001), T stage (HR = 1.445, *p* < 0.001), N stage (HR = 1.443, *p* < 0.001), and risk score (HR = 2.274, *p* < 0.001) were closely related to OS rate of CM patients (Figure 4A). Multivariate Cox regression analysis demonstrated that T stage (HR = 1.396, *p* < 0.001), N stage (HR = 1.654, *p* < 0.001), and risk score (HR = 2.225, *p* < 0.001) were significantly correlated with OS rate for CM (Figure 4B). Subsequently, a model of nomogram was established to accurately predict the 1-, 3-, 5-years OS rate of CM patients based on the ICD-related prognostic signature and clinicopathological characteristics (Figure 4C). Additionally, the calibration curve revealed that the 1-, 3-, and 5-year’s survival time predicted of nomogram exhibited a satisfactory consistency to the actual OS rate for CM patients (Figure 4D). The time-dependent ROC showed that the AUC of 1-, 3-, 5-year was 0.672, 0.660, and 0.661, respectively (Figure 4E). The ROC curves of the training set and validation set was also analyzed to prove the performance of this risk model. (Supplementary Figure S3) Collectively, these results demonstrate that the prognostic signature based on the ICD-related genes is an independent prognostic predictor and accurately estimates the prognosis of CM patients.

### Correlation analysis of immunogenic cell death-related genes prognostic signature and clinicopathological characteristics

**FIGURE 5:**
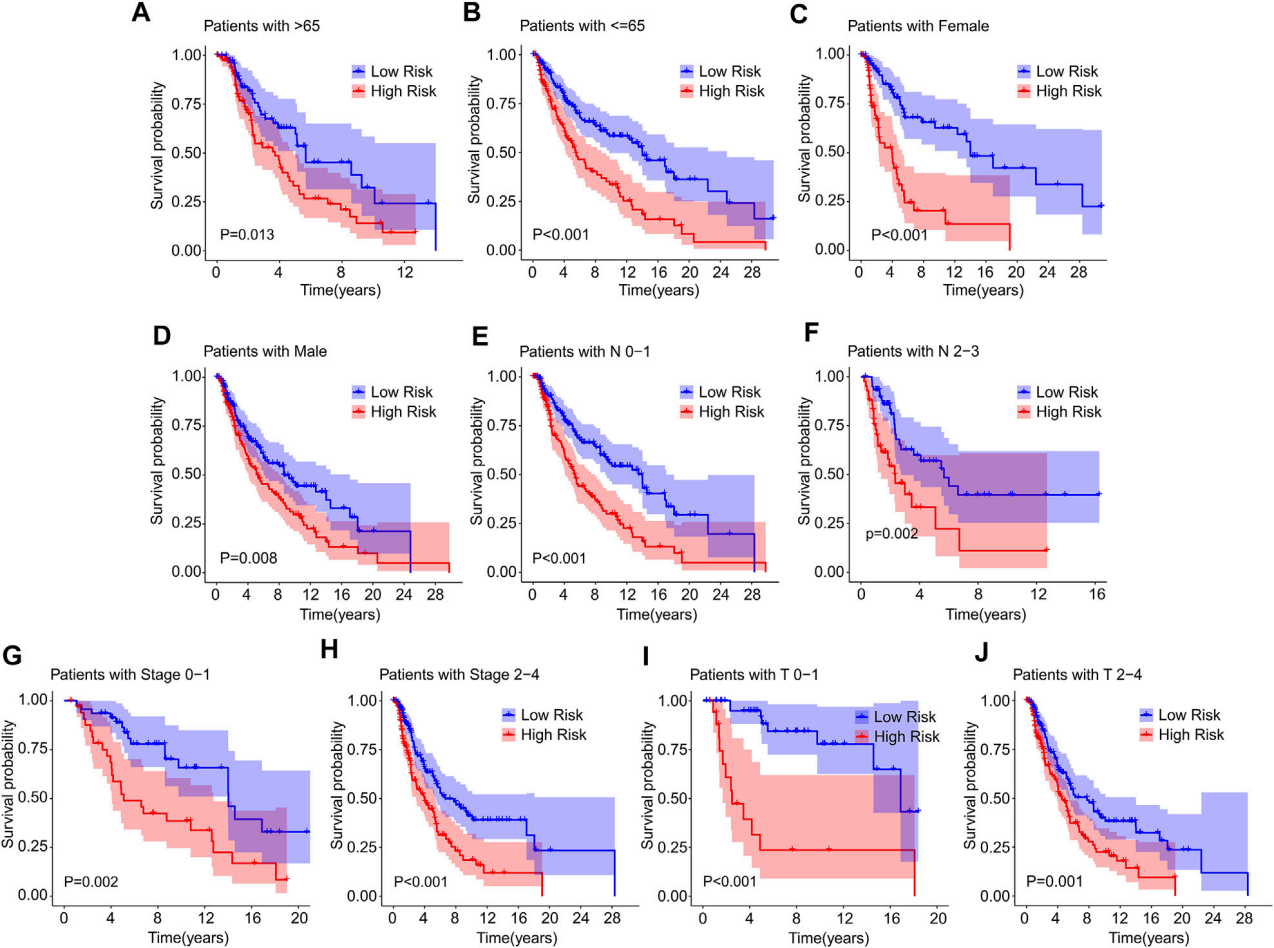
The Kaplan-Meier survival curve of patients in low- and high-risk groups stratified by clinicopathological characteristics. The survival curve analysis reveals the OS rate of patients in low- and high-risk group stratified by **(A,B)** Age (>65 vs. ≤65), **(C,D)** Gender (female vs. male), **(E,F)** N stage (N 0–1 vs. N 2–3), **(G,H)** Stage (stage 0–1 vs. stage 2–4), **(I,J)** T stage (T 0–1 vs. T 2–4).

Thereafter, a stratified subgroup analysis was conducted to investigate the prognostic value of the prognostic signature based on the ICD-related genes. The CM patients were classified into the subgroups according to the age (>65 vs. ≤ 65), gender (female vs. male), N stage (N 0–1 vs. N 2–3), stage (stage 0–1 vs. stage 2–4), and T stage (T 0–1 vs. T 2–4). The Kaplan-Meier survival curve analysis revealed that the OS rate of patients with CM in low-risk group was higher than those patients in the high-risk group based on the ICD-related gene prognostic signature among the different clinicopathological characteristics (Figures 5A–J). These results illustrate that the prognostic signature based on the ICD-related genes could accurately predict the prognosis of CM patients relative to the clinicopathological characteristics.

### Consensus clustering analysis for immunogenic cell death-related genes associated with prognosis and immune infiltration landscape in cutaneous melanoma

**FIGURE 6:**
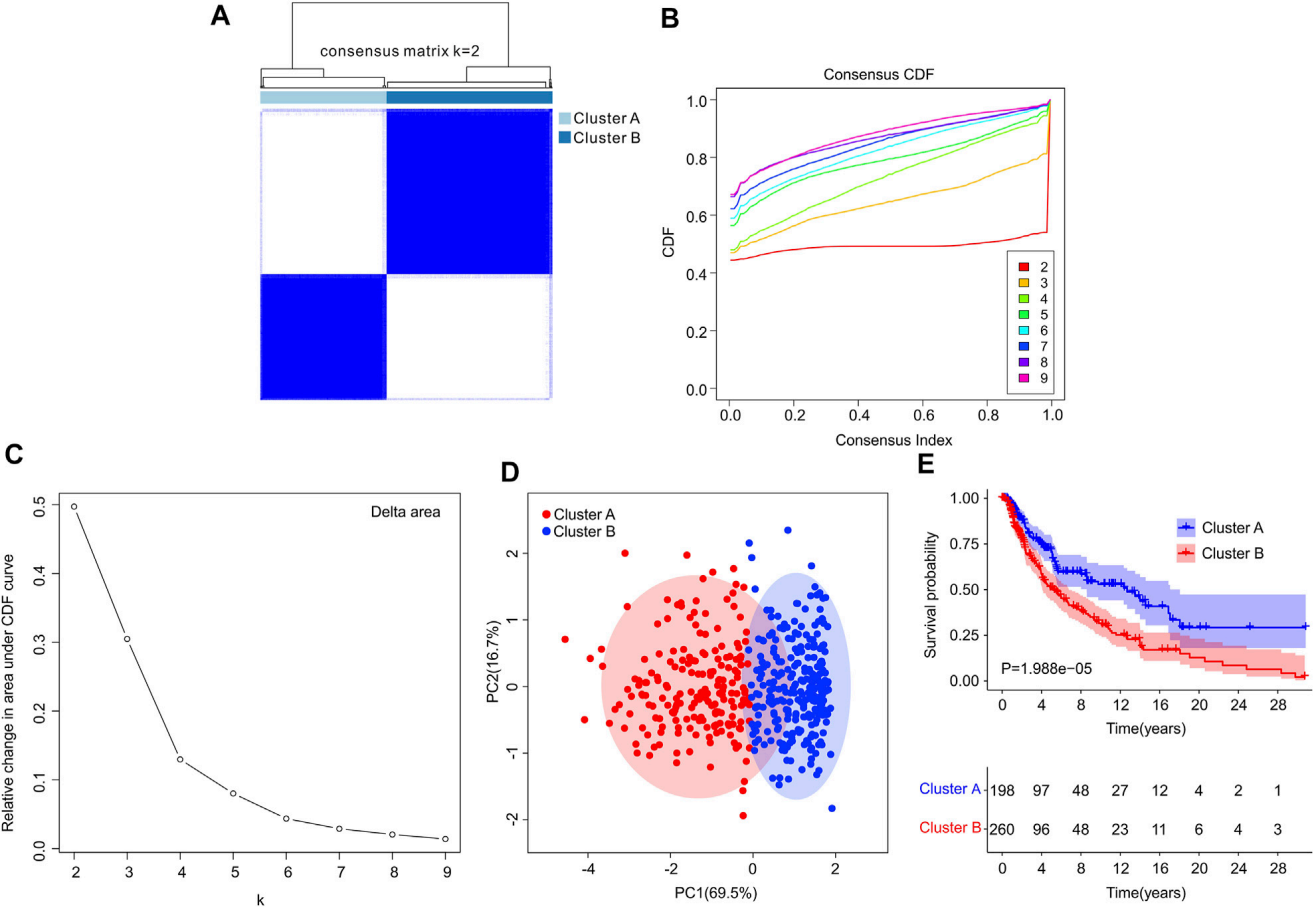
Consensus clustering analysis of CM patients based on the ICD-related genes. **(A)** Consensus clustering heatmap of group at k = 2. **(B)** Cumulative distribution function (CDF) curve for k = 2–9. **(C)** Relative change in area under CDF curve for k = 2–9. **(D)** Principal components analysis (PCA) shows a significant distribution pattern between cluster A and cluster B. **(E)** The Kaplan-Meier survival curve analysis reveals that the OS rate of patients in Cluster A is higher than those in Cluster B.

Consensus clustering analysis was performed to cluster the patients with CM into different subgroup, and the result illustrated an optimal classification for consensus clustering with the *K* = 2 (Figures 6A–C). According to the 4 ICD-related genes, the patients with CM were successfully classified into two subgroups, with 198 patients in Custer A, and 260 patients in Cluster B. The result of PCA showed a clear separation between the Cluster A and Cluster B based on the ICD-related genes (Figure 6D). The Kaplan-Meier survival curve analysis suggested that the patients in Cluster A had higher OS rate than those in Cluster B (Figure 6E).

**FIGURE 7:**
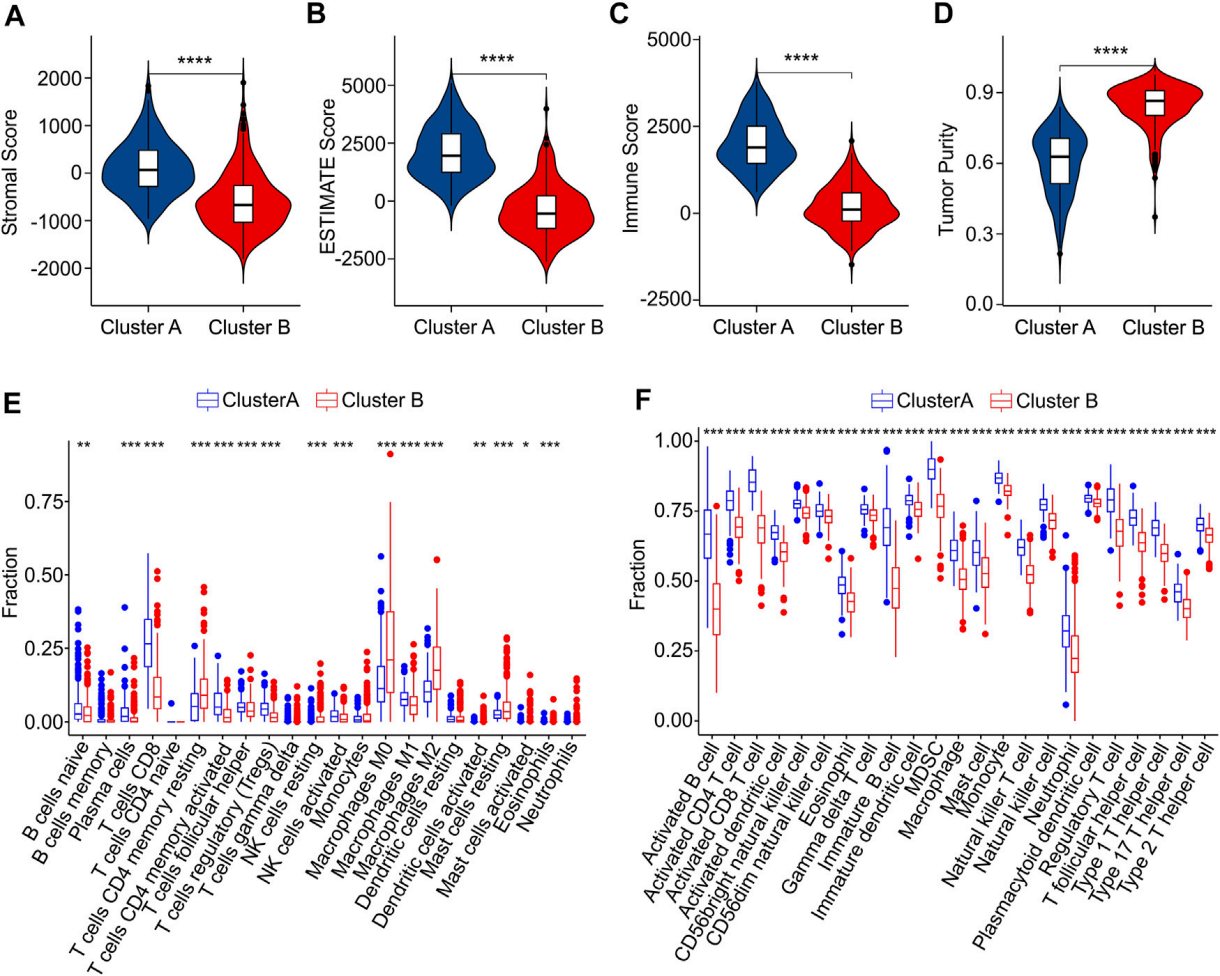
Immune infiltration landscape analysis of CM patients in Cluster A and Cluster B. **(A)** Stromal score. **(B)** ESTIMATE score. **(C)** Immune score. **(D)** Tumor purity. **(E)** The fraction of 22 immune cells in Cluster A and Cluster B calculated by CIBERSORT algorithm. **(F)** The fraction of 23 immune cells in Cluster A and Cluster B *via* ssGSEA algorithm.

Subsequently, multiple immune estimate algorithms were conducted to investigate the immune infiltration landscape of patients in Cluster A and Cluster B. The results of ESTIMATE algorithm showed that the patients in Cluster A had higher stromal, ESTIMATE, and immune score, whereas the tumor purity was significantly higher in Cluster B (Figures 7A–D). To explore the immune infiltration landscape of patients in Cluster A and Cluster B, CIBERSORT and ssGSEA algorithm were performed.

The result of CIBERSORT algorithm illustrated a markedly increased in proportion of B cells naive, plasma cells, CD8 + T cells, CD4 + memory activated T cells and T cells regulatory (Tregs)in Cluster A, but the patients in Cluster B showed a higher proportion of CD4 + memory resting T cells, NK cells resting, macrophages M0, macrophages M0, macrophages M2, dendritic cells activated, mast cells resting, mast cells activated, eosinophils than those patients in Cluster A, indicating a notable difference of immune infiltration landscape in the two subgroups (Figure 7E). Moreover, the result of ssGSEA algorithm revealed that the fraction of 23 immune cells in Cluster A was much greater than in Cluster B, illustrating a higher immune status of patients in Cluster A (Figure 7F). The differential expression of ICD genes between the two clusters was illustrated in the Supplementary Figure S4. These above results demonstrate that the ICD-related genes are associated with the prognosis and immune infiltration landscape of patients.

### The risk model is associated with immune infiltration landscape in cutaneous melanoma

**FIGURE 8:**
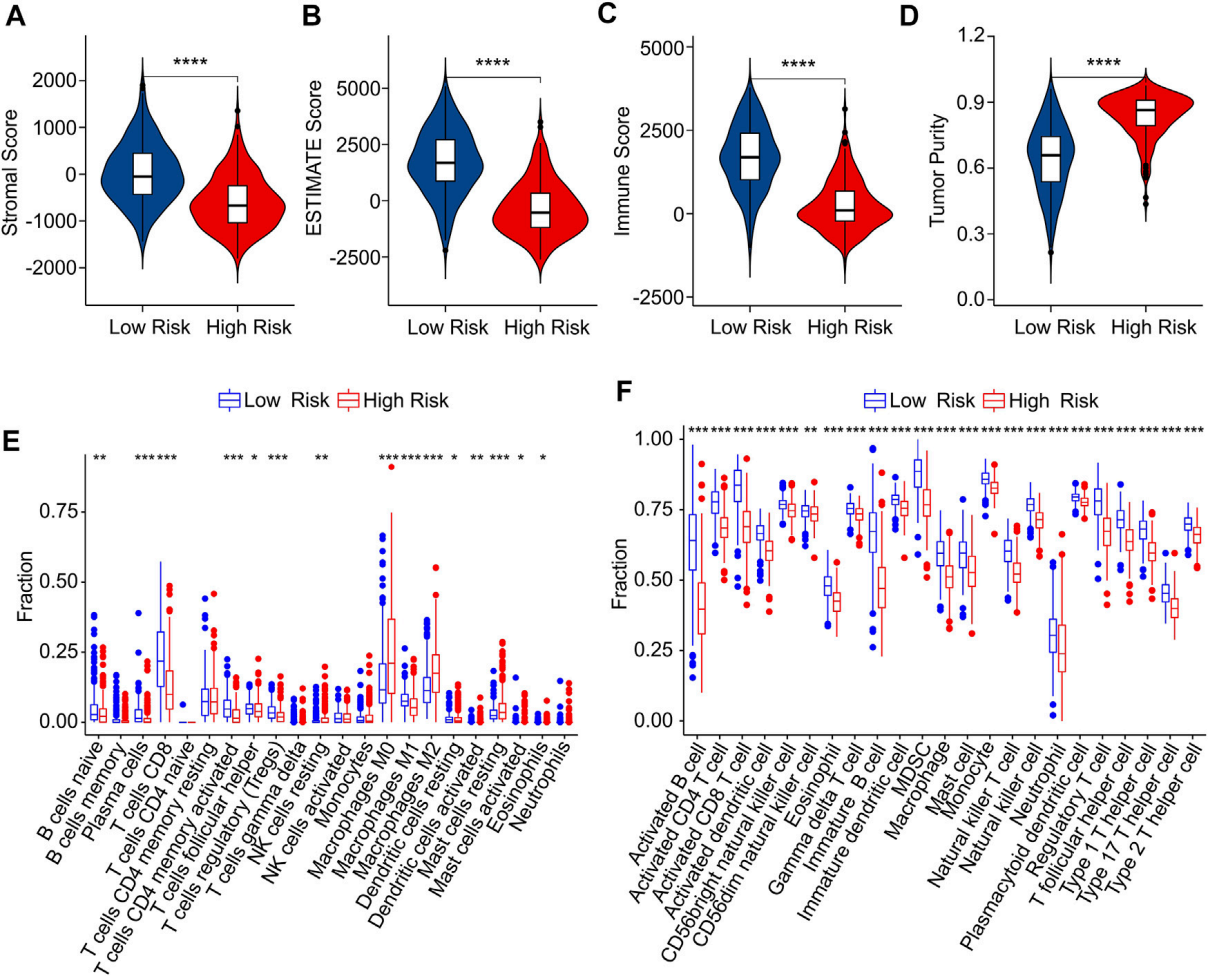
Immune infiltration landscape analysis of CM patients in low- and high-risk group. **(A)** Stromal score. **(B)** ESTIMATE score. **(C)** Immune score. **(D)** Tumor purity. **(E)** The fraction of 22 immune cells in low- and high-risk group calculated by CIBERSORT algorithm. **(F)** The fraction of 23 immune cells low- and high-risk group calculated by ssGSEA algorithm.

The immune infiltration landscape of CM patients in low- and high-risk group was further explored using multiple immune estimate algorithms. The results of ESTIMATE algorithm indicated that the patients with high-risk score had lower stromal, ESTIMATE, and immune scores than those with low-risk score. Notably, the tumor purity in low-risk group was significantly lower than in high-risk group (Figures 8A–D). The result of CIBERSORT algorithm suggested that the fractions of B cells naive, plasma cells, CD8 + T cells, CD4 + memory activated T cells, macrophages M1 and mast cell activated were higher in low-risk group, Inversely, the patients in high-risk group exhibited a markedly increased in the proportion of T cells follicular helper, T cells regulatory (Tregs), NK cells resting, macrophages M0, macrophages M2, dendritic cells resting, dendritic cells activated, mast cells restin, mast cells activated and eosinophils (Figure 8E). The result of ssGSEA algorithm revealed that the fractions of 23 immune cells were significantly lower in high-riak group than in low-risk gorup, indicating that the patients with low-risk score had higher immune status (Figure 8F). Taken together, these findings demonstrate that the risk model for ICD-related gene is associated with the immune infiltration landscape and can indicate the immune status of CM patients.

### Risk model is associated with immunotherapy response

**FIGURE 9:**
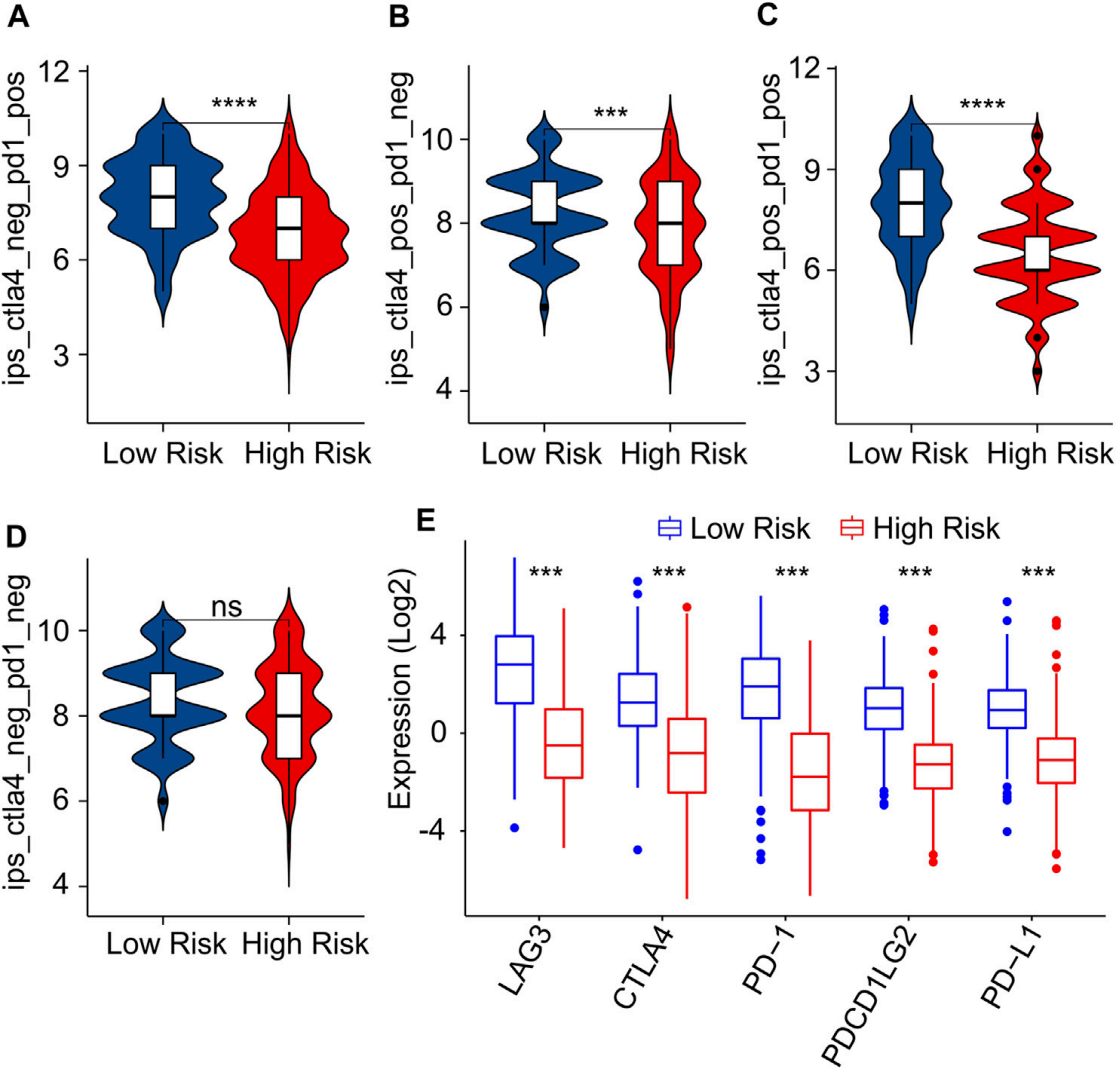
Immunophenoscore (IPS) and immune checkpoint inhibitor (ICI) expression of CM patients in low- and high-risk group. **(A–D)** IPS score shows the response to PD-1 and CTLA-4 for CM patients in low- and high-risk groups. **(E)** Immune checkpoints inhibitor (ICI) expression of patients with CM in low- and high-risk groups.

**FIGURE 10:**
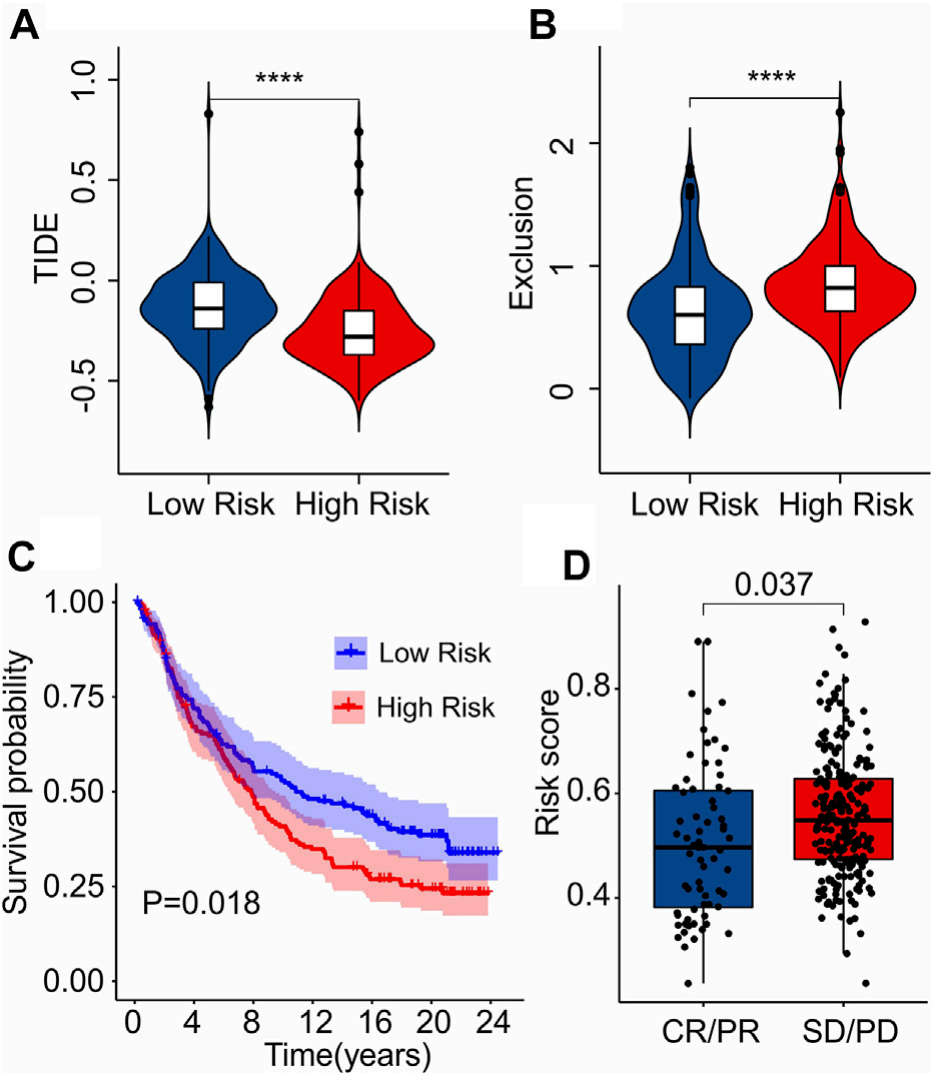
Immunotherapy response analysis. **(A)** TIDE. **(B)** Exclusion. **(C)** The Kaplan-Meier curves shows the OS rate of patients in the low- and high-risk group in anti-PD-L1 cohort (IMvigor210 cohort). **(D)** The risk score in CR/PR and SD/PD indicates a significant difference in the IMvigor210 cohort. PR, Partial Response, PD, Progressive Disease; SD, Stable Disease, and CR, Complete Response.

As a novel predictor of immunotherapy response to anti-*CALT-4* and anti-*PD-1*, immunophenoscore (IPS) has been employed to indicate the response to immune checkpoint inhibitor (ICI) therapy in tumor. Considering the remarkable differences of the immune infiltration landscape in low- and high-risk group, the association between risk score and IPS/ICI was further investigated. The results of IPS analysis revealed that the patients in low-risk group showing a promising response to anti-*CTLA-4*, anti-*PD-1* and anti-*CTLA-4*/anti-*PD-1*, illustrating a better benefit potential in immunotherapy of patients in low-risk group (Figures 9A–D). The result of ICI suggested that the expression of *LAG3*, *CTLA-4*, *PD-1*, *PDCD1LG2*, and *PD-L1* in low-risk group were significantly higher than in high-risk group (Figure 9E). To further illustrate the correlation between the risk score and the efficacy of immunotherapy, IMvigor210 cohort was investigated. Tumor Immune Dysfunction and Exclusion (TIDE) analysis was further applied for the prediction of immunotherapy. According to the prognostic ICD-related genes, the risk score of patients in the IMvigor210 cohort were calculated and divided into the low- and high-risk group. According to TIDE analysis, high-risk patients had a lower TIDE level and a higher exclusion score (Figures 10A,B). The Kaplan-Meier survival curve analysis suggested that the overall survival rate of patients in the low-risk group was significantly higher than patients with high-risk scores (Figure 10C). Additionally, the risk score in CR/PR was significantly lower than in SD/PD in the IMvigor210 cohort, indicating that the patients with low-risk score had a better outcome with immunotherapy (Figure 10D). These results demonstrate a promising immunotherapy sensitivity in low-risk group, providing an innovation insight for the future individualized precision therapy for CM patients in different risk subgroup.

### Correlation analysis of risk score and drug sensitivity

**FIGURE 11:**
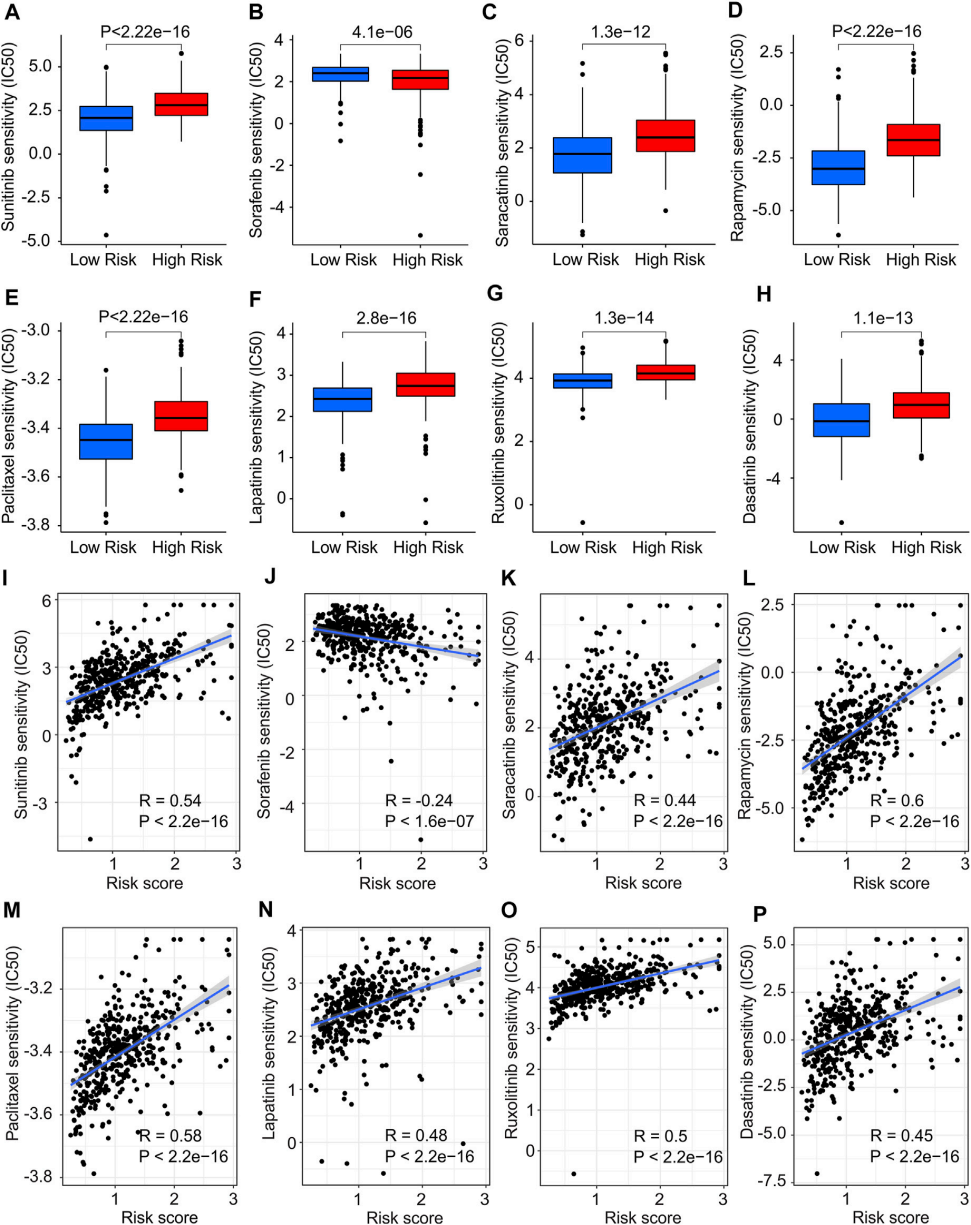
Drug sensitivity analysis in low- and high-risk group. The IC50 value exhibits a significant difference in low- and high-risk group among **(A)** Sunitinib, **(B)** Sorafenib, **(C)** Saracatinib, **(D)** Rapamycin, **(E)** Paclitaxel, **(F)** Lapatinib, **(G)** Ruxolitinib and **(H)** Dasatinib. **(I–P)** Correlation analysis of risk score and drug sensitivity.

The association between the antineoplastic drug sensitivity and risk score was further investigated in the following study. The IC50 of sunitinib, saracatinib, rapamycin, paclitaxel, lapatinib, ruxolitinib and dasatinib in low-risk group were significantly lower than in high-risk group, whereas the IC50 of sorafenib was higher in low-risk group (Figures 11A–H). The correlation of risk score and drug sensitivity indicated that the risk score was significantly positively correlated with sunitinib (R = 0.54, *p* < 2.2e-16), saracatinib (R = 0.44, *p* < 2.2e-16), rapamycin (R = 0.6, *p* < 2.2e-16), paclitaxel (R = 0.58, *p* < 2.2e-16), lapatinib (R = 0.48, *p* < 2.2e-16), ruxolitinib (R = 0.5, *p* < 2.2e-16) and dasatinib (R = 0.45, *p* < 2.2e-16), but negatively correlated with saracatinib (R = -0.24, *p* < 1.6e-07) (Figures 11I–P). These results illustrate a different response of antineoplastic drugs of CM patients in different risk subgroups, suggesting a promising benefit for individualized targeted therapy of CM patients in the future.

### Functional enrichment analysis

**FIGURE 12:**
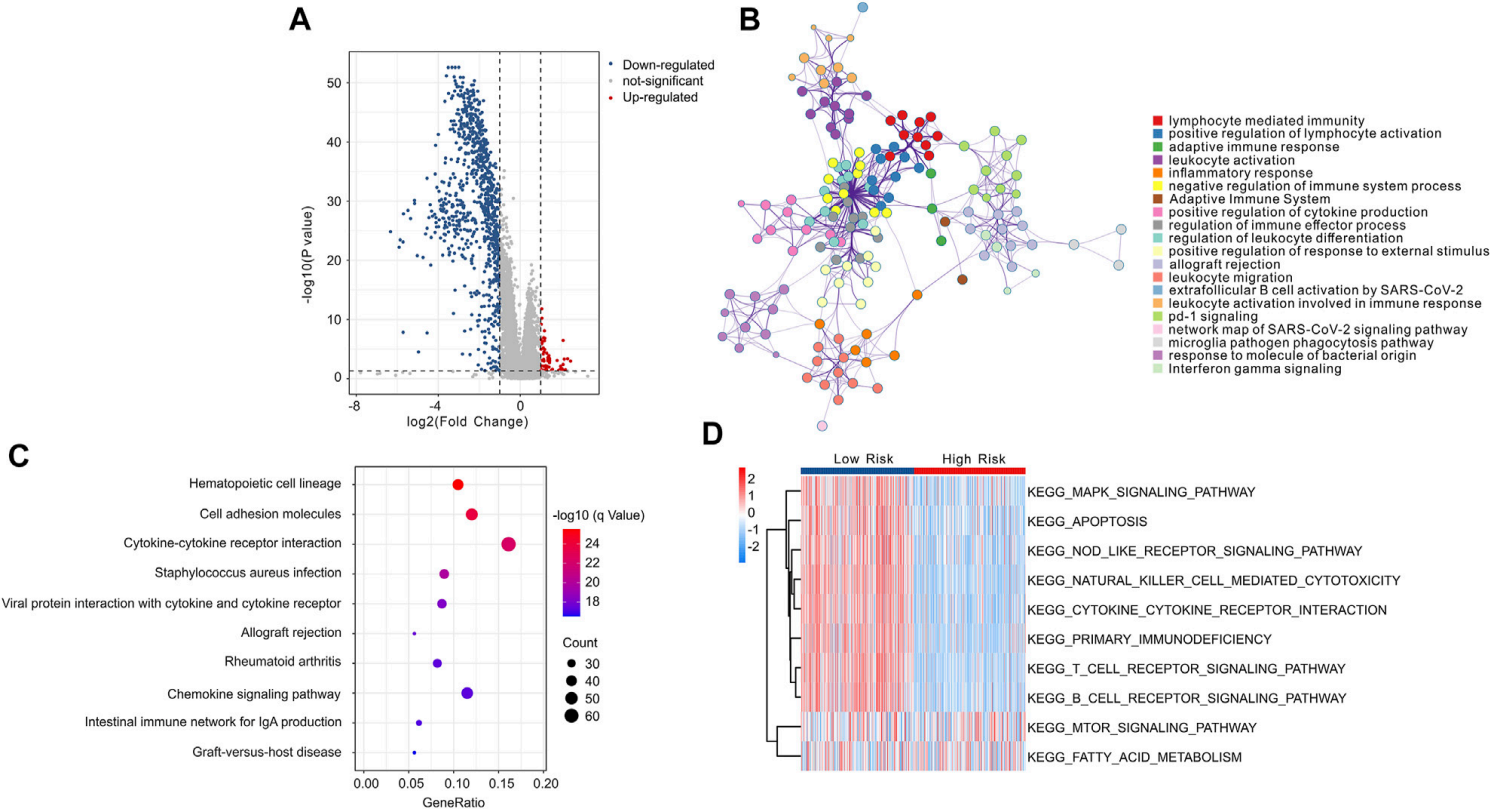
Functional enrichment analysis of differential expressed genes (DEGs) in low- and high-risk group. **(A)** The volcano diagram exhibits the DEGs in low- and high-risk group with the threshold setting at | FC | ≥ 2 and *p*-value < 0.05. **(B)** GO enrichment analysis of DEGs in low- and high-risk groups. **(C)** KEGG enrichment analysis of DEGs in low- and high-risk groups. **(D)** GSVA shows the KEGG terms of each CM patient in low- and high-risk groups.

To explore the potential molecular mechanism associated with the role of ICD-related genes, enrichment analysis and GSVA were utilized. The volcano diagram exhibited the DEGs in low- and high-risk groups, and the result showed that most of the DEGs were down-regulated in high-risk group (Figure 12A). GO enrichment analysis indicated that DEGs were mainly enriched in immune-related procession, such as lymphocyte mediated immunity, and positive regulation of lymphocyte activation (Figure 12B). KEGG enrichment analysis suggested that DEGs were significantly enriched in hematopoietic cell lineage, cell adhesion molecules, and cytokine-cytokine receptor interaction (Figure 12C). Moreover, GSVA analysis was employed to calculate the KEGG terms in each CM patient, and the result showed that immune-related signaling pathways were obviously enriched in low-risk group (Figure 12D). Overall, these findings demonstrate that immune-related processes may mediate the role of ICD-related genes in CM patients.

## Discussion

As one of the most aggressive malignancies, CM takes responsibility for a large proportion of tumor related deaths and the main cause of CM death is early metastasis (Nikolaou and Stratigos, 2014). Therefore, early detection and risk stratification are essential to improve CM survival. In this study, we first constructed a risk model based on four prognostic ICD-related genes, verified its ability to predict the prognosis of CM patients, and preliminarily explored the possible mechanism involved in this process. We also attempted to explore the relationship between prognostic models predicting the prognosis of CM and the immune microenvironment. Considering the heterogeneity of CM tumors, we conducted a consensus clustering analysis based on the ICD genes of the model. By clustering the CM samples, we obtained two subtypes and explored the differences between different subtypes on heterogeneity and tumor microenvironment.

Although melanoma has immunogenicity, it develops an immune escape mechanism to stimulate its rapid progression. These mechanisms include impaired antigen presentation of tumor cells, the accumulation of dysfunctional effector T cells and the production of immunosuppressive TME (Oliveira et al., 2021). Therefore, many methods have been developed to revitalize the anti-tumor immune response. Recently approved immune checkpoint inhibitors (ICI) immunotherapies have completely changed the treatment of CM with significantly improved survival rate and disease lasting control (Hodi et al., 2018; Hamid et al., 2019). However, the response rate to ICI is still limited (Reijers et al., 2022). Therefore, further efforts should be made to maximize the efficacy of ICI treatment. ICD targeting has been proved to be an effective way to prevent CM carcinogenesis (Fu et al., 2022; Zhang et al., 2022). In our data, TIDE analysis between different groups was used to predict the effect of immunotherapy. The low-risk group was more responsive to immunotherapy. Combined with the significance of immunotherapy in clinical application, the influence of ICD classification on prognosis was explained. In addition, differences in drug sensitivity as determined by ICD may also partly account for differences in prognosis. This is in line with the report that ICD related to CM immunophenotype cold to hot transformation. In some CM patients, adverse tumor microenvironment (TME), lack of invasive T lymphocytes, or increased Tregs failed to respond to ICI. This kind of situation is called “cold” TME. The latest report says that by activating ICD, targeting wee1/akt pathway can lead to the recruitment and activation of immune cells in TME, triggering an inflammatory cascade, so that the “cold” TME of melanoma can be transformed into a “hot” TME responsive to programmed cell death proteins, leading to the complete regression of established tumors (Dinavahi et al., 2022). Combined with the significant prognostic significance of ICD-related genes risk model in this paper, further research on the process of ICD will help to achieve a better clinical prognosis of CM.

As a member of the Bcl-2 family, *BAX* forms pores in the outer membrane of mitochondria, resulting in the release of pro-apoptotic factors into cytosol, thus initiating the process of apoptosis (Tait and Green, 2010). In CM, low expression of *BAX* has been reported to be associated with higher PFS (Gutta et al., 2020). The correlation between higher *BAX* expression and poor prognosis in our high-risk group also verified this conclusion. This is not consistent with the intuitive role of *BAX* in promoting apoptosis in tumors. In fact, in acute myeloid leukemia and non-Hodgkin lymphoma, highly expressed *BAX* is also associated with poor outcome. Since the mechanism is not fully elucidated, there is therefore a need to interpret or study characteristics that indicate apoptosis capacity or resistance in specific disease settings and contexts (Gutta et al., 2020). It has been proposed that following effective apoptosis-induced therapy, dormancy in tumor tissue, stem-cell-like cancer cells repopulate the tumor and promote further spread and progression of the disease after amplification (Labi and Erlacher, 2015; Ichim and Tait, 2016). This may partly explain why expression patterns of high apoptotic reactivity are associated with poor prognosis. Chemokine receptor *CXCR3* has been reported to be a biomarker of sensitivity to PD-1 blockade (Chow et al., 2019; Telli et al., 2021). Combined with the important role of PD-1-related therapy in CM, the correlation between higher *CXCR3* expression level and better prognosis of CM can be explained. In addition, the important role of *CXCR3* in CM T cell transport was also noted (Mikucki et al., 2015). This is consistent with our results that higher *CXCR3* expression levels are associated with higher T cell infiltration levels in TME. It was previously thought that melanoma inhibits the killing effect of the immune system by secreting immunosuppressive cytokines including IL-10 (Chen et al., 1994). However, as reviewed in 2019, IL-10 showed conflicting effects on immunity and cancer (Ouyang and O'Garra, 2019). IL-10 itself has an effective anti-tumor effect and also inhibits metastasis through immune-dependent mechanisms, including inhibition of infiltrating macrophages and angiogenic factors and activation of CD8^+^ T cell CTL (Berman et al., 1996; Fujii et al., 2001; Mannino et al., 2015). In addition, IL-10 can activate CD4^+^ T cells and CD8^+^ CTLs under certain *in vitro* conditions (Groux et al., 1998). This is consistent with our data that the low-risk group with higher IL-10 expression level has a better prognosis and the corresponding results of immune infiltration. Our data suggest that low *EIF2AK3* gene expression levels in this cohort are associated with poorer outcomes. As an unfolded protein response (UPR) protein kinase, *EIF2AK3* (also known as PERK) regulates protein synthesis. Although *EIF2AK3* has been reported to be necessary for the progression of CM, it also has the ability to trigger pro-apoptotic signals and inhibit cell division by inhibiting cyclin D1 translation in CM (27977682). In addition, *EIF2AK3* induces immune metabolic reprogramming and enhances anti-tumor T cell function (Chakraborty et al., 2022). Therefore, the dual characteristics of tumor inhibition and tumor promotion of *EIF2AK3* still need to be further studied in CM.

In our results, lower CD8^+^ T, CD4^+^ T cell, B cell, plasma cell, MDSC cell level and higher M2 macrophage cell level indicated that patients in the high-risk CM group undoubtedly had immunosuppressive TME. The significance of DAMP in TME has been reported. ATP, as a DAMP member, directs immune cells to inflammatory sites; in addition, the loss of its receptor almost completely blocks macrophage activity and accumulation of CD4^+^ T and B cells (Merz et al., 2018). Moreover, ATP from dying cancer cells promotes proteolytic maturation of caspase-1 and cleavage and release of interleukin (IL)-1β (Ghiringhelli et al., 2009). In addition, as an important member of DAMP, the CALR molecule acts as an important “eat me” signal against the “don’t eat me” signal of tumor cells to promote antigenic uptake and immune recognition of APC (Le Saux et al., 2021). *HMGB1* was characterized extracellular as a pro-inflammatory predictor. As a “danger” signal, *HMGB1* polarizes pro-inflammatory microglias through the RAGE-NF-κB pathway, thereby activating innate immunity (Fan et al., 2020). After ICD development in tumor cells, *HMGB1* acts on TLR4 on DC and promotes optimal processing of tumor antigen toward crossover triggering T cells (Moriya et al., 2021). Our data again validated the significant correlation between ICD-related TME changes and CM prognosis.

In conclusion, we constructed a risk model consisting of 4 ICD-related genes and effectively predicted the prognosis of CM patients. We also comprehensively analyzed the immune microenvironment between high and low risk groups. The correlations of immune infiltration level, immune response and drug sensitivity treatment between two risk levels were further explored and signal pathways involved were preliminarily analyzed. This study provides a new perspective and insight for individualized and accurate treatment strategies for CM patients.

## Materials and methods

### Ethics statement

This study has been approved by the Ethics Committee of Qilu Hospital (Jinan, China). The data was retrieved from published literature, and all analysis were performed in accordance with the Declaration of Helsinki.

### Transcriptome data and clinical data collection

The normalized transcriptome gene expression matrix (RNA-Sep, FPKM format) and clinical information materials were downloaded from The Cancer Genome Atlas database (TCGA) (https://portal.gdc.cancer.gov/). The transcriptome gene expression matrix of the normal tissues for CM were downloaded from the UCSC Xena database (https://xenabrowser.net/datapages/) (RNA-Sep, FPKM format). Moreover, the normalized transcriptome gene expression matrix of normal tissues and tumor tissues was merged and normalized for the subsequent analysis. The samples without survival time were excluded and a total of 458 CM samples were included for the subsequent analysis. Perl scripts were conducted to merge the gene expression matrix of each sample and the expression of mRNAs were annotated using the ensembles human genome browser GRCh38.p13 (http://asia.ensembl.org/index.html). The transcriptome matrix of GSE65904 was obtained from the GEO database (https://www.ncbi.nlm.nih.gov/geo/) and we extracted the expression file from above transcriptome matrix via Perl scripts. A total of 210 CM samples were collected from GSE65904 for the further analysis. The transcriptome data of TCGA and GEO were merged and removed batch effects via “SVA” R package. The clinical information materials included survival time, survival status, age, gender, stage, and T, N stage were obtained using Perl scripts from the TCGA database. In this study, all information and clinical matrix involved were downloaded from the public database. Approval from the ethics committee and written informed consent from patients were not required.

### Risk model construction of immunogenic cell death-Related genes

The Immunogenic Cell Death (ICD) related genes were identified and extracted from the previous research, and a total of 33 ICD-related genes were included to construct the risk model (Garg et al., 2016) (Supplementary Table S1). Based on univariate Cox regression analysis, the least absolute shrinkage and selection operator (LASSO) algorithm was employed to identify the ICD-related genes associated with overall survival (OS) rate using R package “glmnet”. Then, multivariate Cox regression analysis was performed to identify the candidate prognostic ICD-related genes and constructed the risk model. The risk score of each sample was calculated according to the following formula: = (−0.244 x the expression of CXCR3) + (−0.236 x the expression of IL10) + (−0.344 x the expression of EIF2AK3) + (0.276 x the expression of BAX expression). Thereafter, the CM patients were divided into low- and high-risk groups according to the median risk score. The Kaplan-Meier survival curve was conducted to estimate the OS rate of patients in low- and high-risk group via log-rank algorithm using R packages “survival”. The principal component analysis (PCA) was used to investigate the separation pattern of patients in low- and high-risk group based on the prognostic ICD-related genes using R package “ggplot2”.

### Validation of the risk model

According to the ICD-related genes, the samples in TCGA database were classified into the training cohort and the test cohort to the ratio of 7:3, with 321 samples in the training cohort and 137 samples in the test cohort, and calculated the risk score of each sample, respectively. Moreover, GSE65904 was utilized to validate the stability of the risk model as an external validation cohort. The risk score of each sample was calculated and divided into low- and high-risk groups according to the median risk score.

### Independence evaluation of risk model

Univariate and multivariate Cox regression analysis were employed to investigate the risk model was an independent indicator for CM using R package “survival”. A nomogram model was constructed of clinicopathological characteristic and risk score via R package “rms”. According to Cox regression analysis, all variates were calculated and estimated the 1-, 3- and 5- year’s survival probability of patients. Calibration diagram and consistency index (C-index) were commonly parameters to assess the accuracy of nomograms and the C-index was positively correlated with the nomogram accuracy. The prognostic capability of the risk model constructed by risk score was validated using time-dependent receiver operating characteristic (ROC) analysis via R package “timeROC”.

### Consensus clustering

According to the prognostic ICD-related genes, consensus clustering was performed using the R package “ConsensusClusterPlus”. The clustering was established on the grounds of partitioning around medoids with “Euclidean” distances and 1,000 verifications were performed. Finally, according to the optimal classification of K = 2−9, the patients with CM were clustered into two subtypes for the further analysis.

### Immune infiltration landscape analysis

ESTIMATE algorithm was conducted to evaluate the estimation of stromal and immune cells in tumor. Stromal, immune, ESTIMATE score, and tumor purity were calculated via R package “estimate”. CIBERSORT algorithm was utilized to investigate the immune infiltration landscape, and 22-types immune cells were evaluated based on “CIBERSORT R script v1.03”. A single sample gene set enrichment analysis (ssGSEA) algorithm was performed to assess the proportion of 23-types of immune cells via the “GSVA” R package.

### Immunotherapy response and drug sensitivity analysis

In this study, the result of Immunophenoscore (IPS) was obtained from the TCIA database (https://tcia.at/home). The expression of immune checkpoint inhibitor (ICI) included *LAG3*, *CTLA4*, *PD-1*, *PDCD1LG2*, and *PD-L1* were extracted from the TCGA matrix using R package “limma”. The expression of ICI was transformed by log_2_(expression + 1). Tumor Immune Dysfunction and Exclusion (TIDE) Analysis was analyzed using TIDE database (http://tide.dfci.harvard.edu/login/). An anti-PD-1/PD-L1 treatment cohort (IMvigor210) cohort was used to evaluate the response of anti-PD1/PD-L1 for CM patients. The expression of ICD-related genes was extract from the IMvigor210 cohort and the risk scores of each sample were calculated. A total of 348 samples were divided into low- and high-risk group. Drug sensitivity (IC50) was a vital indicator for evaluating drug efficacy or sample response to treatment. Based on the Genomics of Drug Sensitivity in Cancer (GDSC) database, the drug response of each sample in low- and high-risk was predicted via R package “pRRophetic”. All statistical analyses were visualized via “ggplot2” R package.

### Functional enrichment analysis

The R package “limma” was used to identify the differential expressed genes (DEGs) in low- and high-risk group, and the *p*-value was adjusted using “FDR” method. Moreover, the threshold for screening DEGs was set at |Fold Change| ≥ 2 and *p*-value < 0.05. Metascape database (http://metascape.org/) was used to explore the potential biological functions of DEGs, and Kyoto Encyclopedia of Genes and Genomes (KEGG) analysis was performed to enrich the DEGs into pathways using the “clusterProfiler” R package (Yu et al., 2012). The activity of KEGG term in each patient with CM was conducted using R package “GSVA”.

### Statistical analysis

All statistical analyses were performed using R software (version 4.1.0) and Perl scripts. Spearman-ranked correlation analysis was applied to investigate the correlation between risk score and IC50, with *p*-value < 0.05 was considered significantly different. Differential functions were analyzed using the Wilcoxon rank-sum test between the two groups, and statistical significance was set at *p*-value < 0.05.

## Data Availability

The datasets presented in this study can be found in online repositories. The names of the repository/repositories and accession number(s) can be found in the article/Supplementary Material.
