# Supplementary figures and images for: Significance of immunogenic cell death-related genes in prognosis prediction and immune microenvironment landscape of patients with cutaneous melanoma
Source: Front Genet. 2022 Sep 21;13:988821. doi: 10.3389/fgene.2022.988821 (PMC9532744; doi:10.3389/fgene.2022.988821)

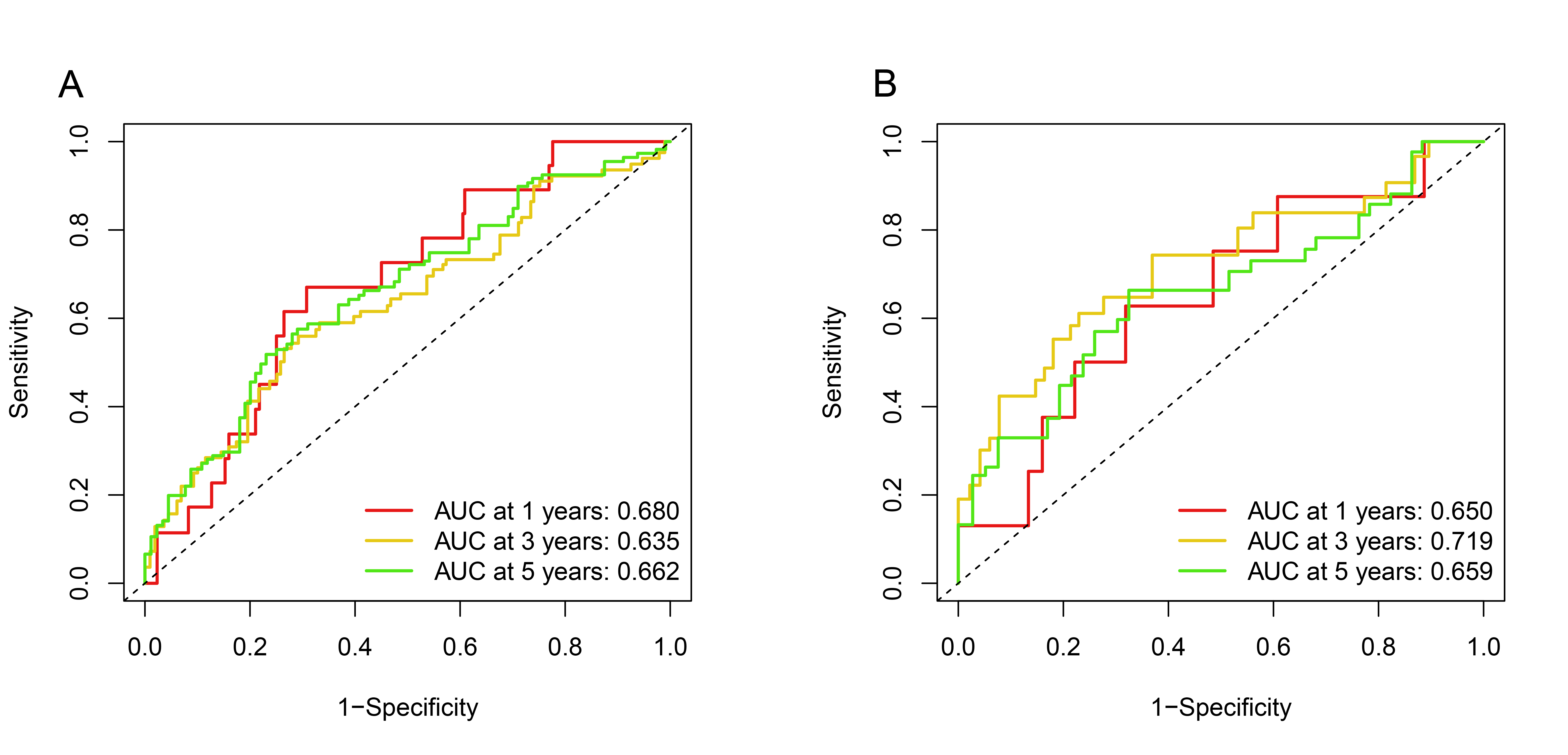

Supplement: Supplementary file 1 [file Image3.JPEG]

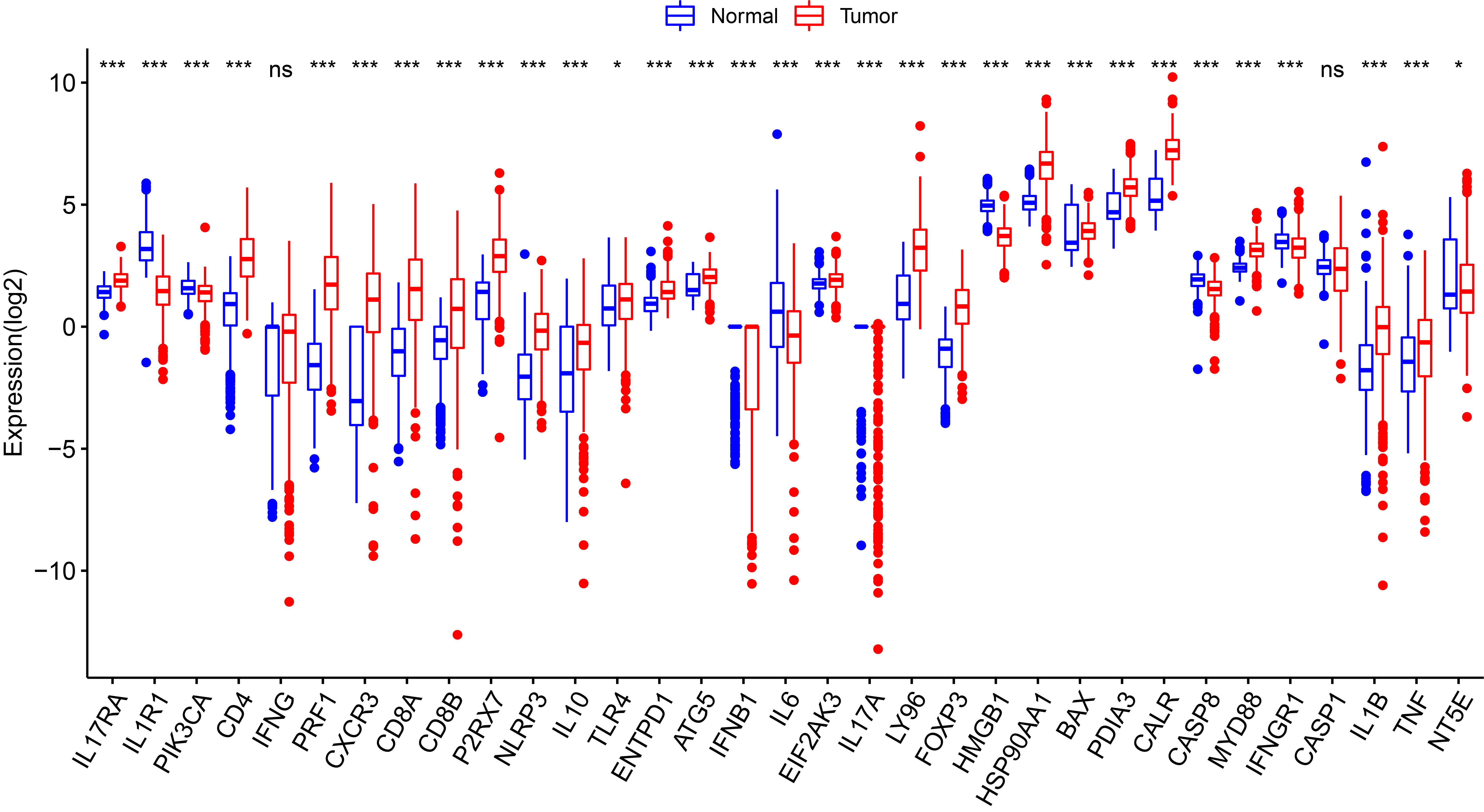

Supplement: Supplementary file 2 [file Image1.JPEG]

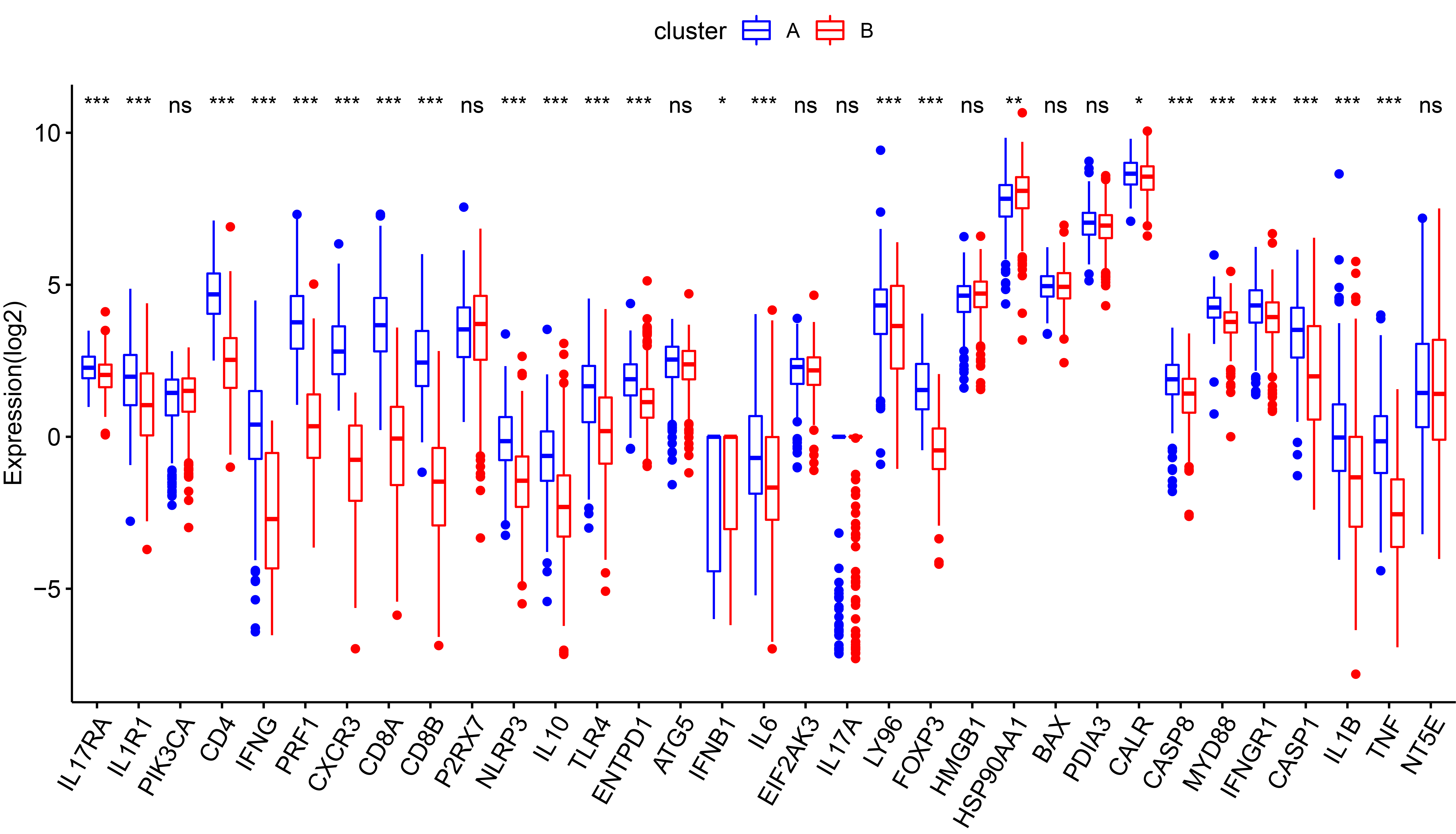

Supplement: Supplementary file 3 [file Image4.JPEG]

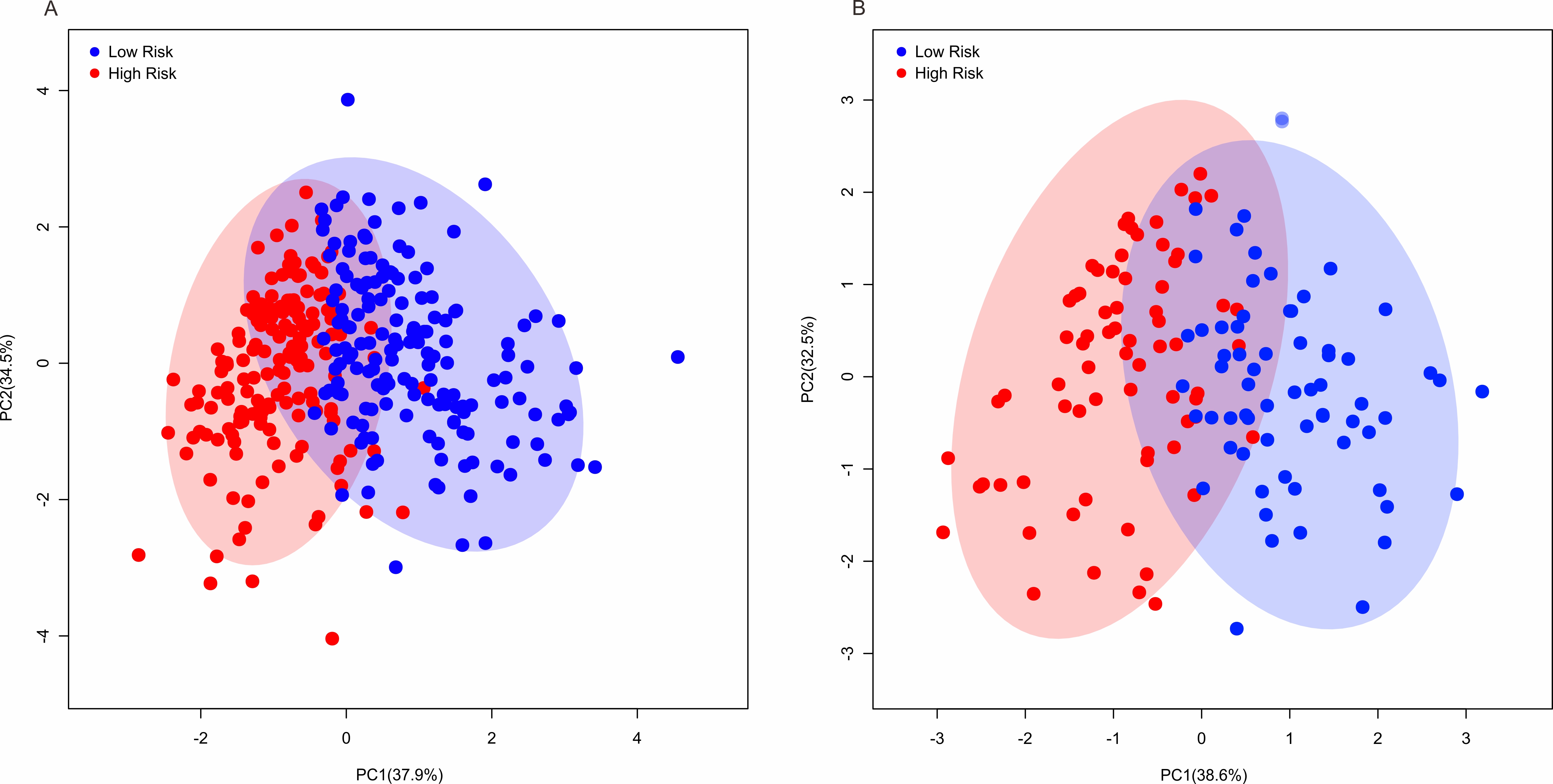

Supplement: Supplementary file 4 [file Image2.JPEG]
