# Supplementary material for: Significance of immunogenic cell death-related genes in prognosis prediction and immune microenvironment landscape of patients with cutaneous melanoma
Source: Front Genet. 2022 Sep 21;13:988821. doi: 10.3389/fgene.2022.988821 (PMC9532744; doi:10.3389/fgene.2022.988821)
Supplement: Supplementary file 5 [file Table2.DOCX]

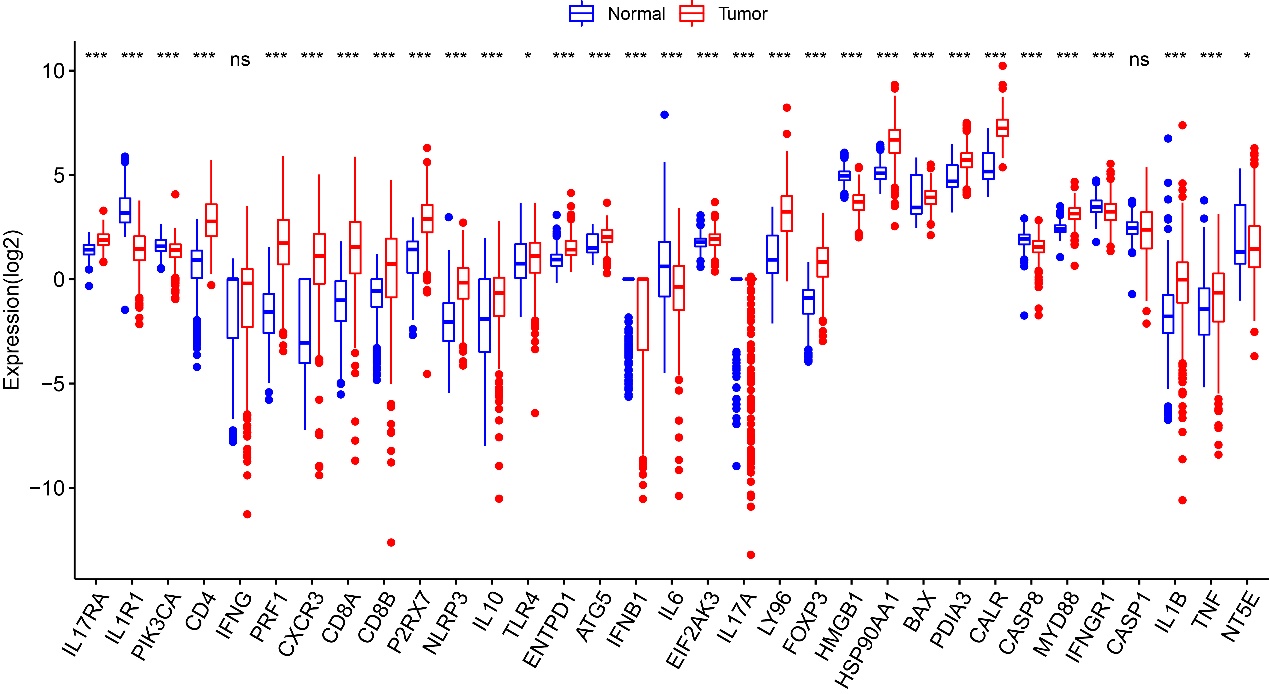


Supplementary Figure 1. The expression of ICD-related genes in normal tissues and tumor tissues. The expression data is transformed by log_2_(expression + 1).


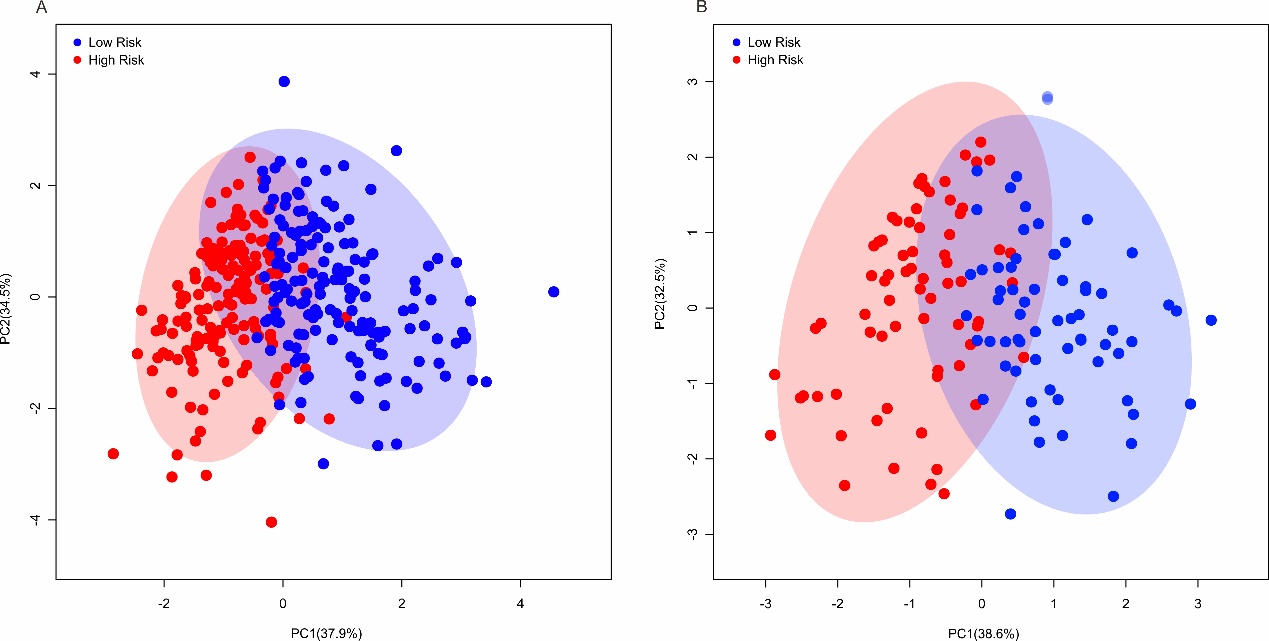


Supplementary Figure 2. Principal comment analysis (PCA) training cohort and test cohort. The result of PCA shows a significant difference between the low- and high-risk group based on the 4 prognostic ICD-related genes in (A) training cohort and (B) test cohort.


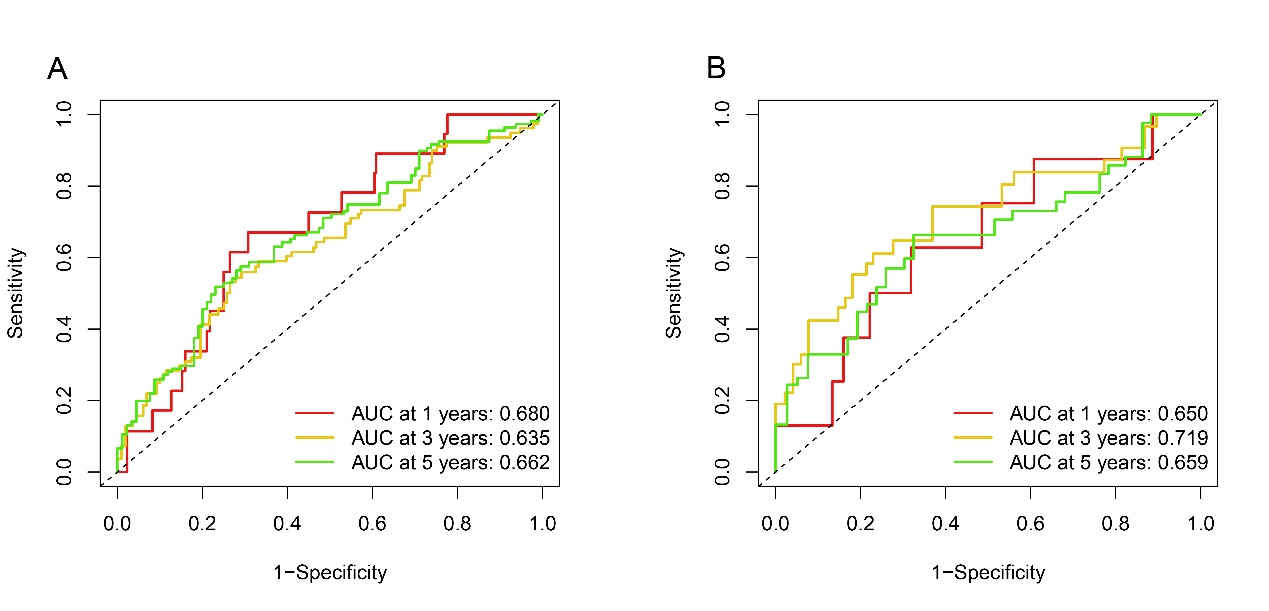


Supplementary Figure 3. Time-dependent ROC curve shows the AUC at 1-, 3-, and 5-years in training cohort (A) and test cohort (B).


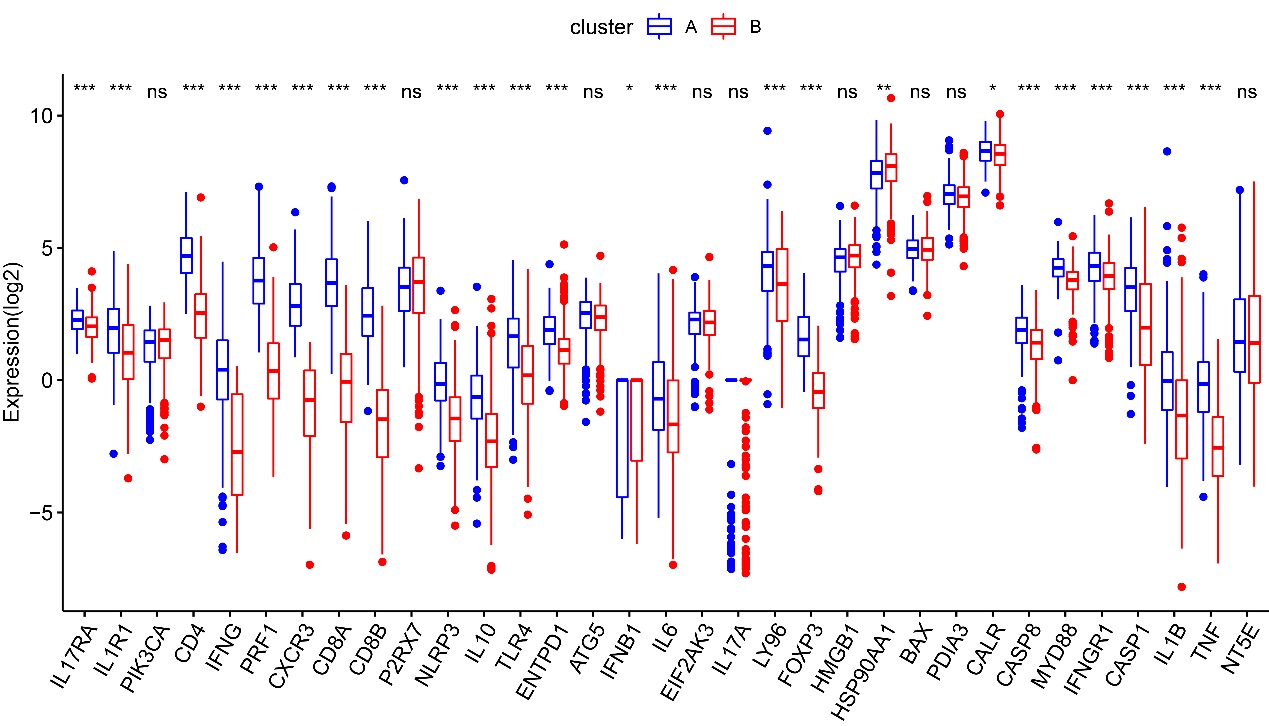


Supplementary Figure 4. The expression of ICD-related genes in Cluster A and Cluster B. The expression data is transformed by log_2_(expression + 1).
